# Electrospinning of Well‐Aligned P(VDF‐TrFE) Fibers Using a Benign Solvent

**DOI:** 10.1002/marc.202500099

**Published:** 2025-03-29

**Authors:** Shaashwat Saraff, Kalyan Ghosh, Thiyagarajan Natarajan, Giulio Isacco Lampronti, Sohini Kar‐Narayan

**Affiliations:** ^1^ Department of Materials Science and Metallurgy University of Cambridge 27 Charles Babbage Road Cambridge CB3 0FS United Kingdom

**Keywords:** alignment quality metrics, electrospinning, functional polymers, green solvents, health and safety, P(VDF‐TrFE), piezoelectricity

## Abstract

Poly(vinylidene fluoride‐co‐trifluoroethylene) (P(VDF‐TrFE)) is an important piezoelectric polymer, often electrospun into fibrous membranes for technological applications. Typically, this involves toxic solvents, necessitating a compromise between solvent safety, process stability, and fiber quality. In this study, out of several candidates across different organic families, a safe and effective solvent is identified for electrospinning P(VDF‐TrFE). The dual impact of solvent boiling point on process stability and fiber quality is studied, to arrive at a facile, consistent protocol for producing high‐quality fibers. The health effects of the solvents are considered, as those posing minimal risks are favored in applications related to biomedicine, for example. Methyl propyl ketone (MPK) is found to be an ideal, non‐toxic solvent, with an optimal evaporation rate at typical processing temperatures, to produce uniform, distinct and well‐aligned electrospun P(VDF‐TrFE) fibers. The impact of electrospinning process parameters is further investigated to optimize results using this solvent. Through the introduction of a benign and effective solvent for electrospinning, this work provides a detailed protocol to produce high‐quality P(VDF‐TrFE) fibers through a consistent and stable process, without involving toxic solvents, which opens up new possibilities in green electrospinning.

## Graphical abstract

The role of solvent properties is explored in electrospinning a piezoelectric polymer popular in electromechanical devices. The relationship between solvent boiling point, process stability and fiber quality is viewed through the lens of morphological and crystalline phase characterization. Solvent properties and process parameters are optimized to arrive at a facile protocol for electrospinning P(VDF‐TrFE) while minimizing biological and environmental risks.

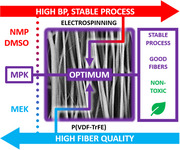

## 1 Introduction

Piezoelectric polymers form an active area of research, with wide‐ranging applications including tissue engineering, electromechanical energy harvesting, sensing, and actuation.^[^ ^1^, ^2^, ^3^ ^]^ Typically, in order to make a device out of them, they must be processed through techniques such as stretching, annealing or poling to enable their piezoelectric behavior,^[^ ^1^ ^]^ and they must be formed into the device component using an appropriate fabrication technique. Electrospinning is a technique that can combine these two functions in a single step, as the in situ electric field acts to transform the polymer into its piezoelectric form, while the process overall produces a 2D membrane of polymer fibers that can be integrated as‐is into the device. Mechanical stimuli land on this 2D membrane, and give rise to electrical signals in the piezoelectric polymer that can be picked up by the peripheral circuitry forming the rest of the device. Piezoelectric fibers thus formed have been incorporated into energy harvesting^[^ ^4^, ^5^, ^6^ ^]^ and sensing^[^ ^7^ ^]^ devices as well as tissue scaffolds.^[^ ^8^ ^]^ Electrospinning involves a polymer melt or solution being fed into a nozzle, pointed toward a collector at a controlled separation, each kept at a set voltage, resulting in an electric field in the gap. This field causes a jet of liquid to rush from the nozzle, and in‐flight cooling or drying solidifies this into fine fibers of the polymer, which are deposited on the collector. Compared to melt electrospinning, solution electrospinning is more widely studied, and preferred for its ability to achieve finer fibers while employing a simpler setup without necessitating a heated liquid reservoir.^[^ ^9^ ^]^

The theory of Hansen solubility parameters (HSPs) provides a helpful framework for understanding and working with polymer solutions. The parameters $(\delta_{D},\delta_{P},\delta_{H})$ capture an organic substance's tendency to interact with others through dispersion, polar, and hydrogen‐bonding forces respectively, and together define a 3D space in which the substance (polymer or solvent) may be located as a point. The “Hansen distance” $D_{12}$ between two substances with HSPs $(\delta_{D1},\delta_{P1},\delta_{H1})$ and $(\delta_{D2},\delta_{P2},\delta_{H2})$ is mathematically defined through Equation 1, and is a measure of their mutual dissimilarity in the context of mixing. Some calculated examples are presented in Table S1 (Supporting Information). A polymer usually has a threshold distance, such that a solvent well within that distance from the polymer in Hansen space will dissolve it well, a solvent close to the threshold will barely dissolve (or only tend to swell) the polymer, and a solvent beyond this distance will fail to dissolve it. An important and advantageous consequence of the theory of HSPs is the “lever rule”, which states that the effective HSPs of a solvent blend are equal to the average of the HSPs of the individual solvents, weighted by their volumes. This allows the formulation of bespoke solvent blends to closely match the HSPs of a target polymer, when no single solvent is available for dissolving it, or other considerations (e.g. cost, safety, etc.) preclude suitability.^[^ ^10^, ^11^, ^12^ ^]^

(1) $$D_{12}=\sqrt{4{\left(\delta_{D1}-\delta_{D2}\right)}^{2}+{\left(\delta_{P1}-\delta_{P2}\right)}^{2}+{\left(\delta_{H1}-\delta_{H2}\right)}^{2}}$$

In a detailed study^[^ ^13^ ^]^ on the electrospinning of poly(lactic acid) (PLA), the authors have investigated the effect of solvent boiling point (BP) on process stability. They found that low‐boiling solvents such as dichloromethane (DCM) (BP: 40 °C) evaporated prematurely, leaving a dried polymer residue at the nozzle tip that caused it to get blocked within a few minutes of starting the electrospinning process. This constant interruption to the process resulted in the need to clean the nozzle every few minutes in order to continue deposition. Linking this problem to our preceding discussion, we observe that the Hansen framework can guide the selection of a suitable alternative solvent in such situations, even without accurate knowledge of the HSPs of the polymer. If a solvent is known to have desirable chemical properties (i.e., good solvency for the target polymer) but non‐ideal physical properties (e.g. BP being too low), the new solvent candidate may be chosen such that it has better‐suited physical properties while still having HSP values similar to the old solvent, meaning that it would still be likely to dissolve the polymer but might offer greater electrospinning process stability. Although keeping the low‐/high‐BP solvent unchanged and instead adjusting the ambient parameters (e.g. temperature, humidity, solvent vapor pressure, etc.) within the electrospinning chamber may be another solution, this is in practice quite challenging, since these parameters require expensive hardware to control and need a long time to re‐stabilize after any perturbations to the system, such as opening the chamber. Moreover, using the process temperature to control evaporation rate (rather than choosing a solvent of a different BP) may not always work as expected due to temperature‐dependent changes in the viscosity of the solution which would affect electrospinning/electrospraying behavior and the resultant product properties. Thus, finding a solvent that behaves as desired under standard laboratory conditions is often the best solution.

Poly(vinylidene fluoride) (PVDF) is a popular functional polymer used in electromechanical devices due to its piezoelectric nature. It has several crystalline phases, of which the β phase, comprising all‐trans chains, is the most desirable as it has the highest net polarization, leading to optimal piezoelectric performance. Other phases may be transformed into the β phase through manipulations such as annealing, stretching and poling (i.e., applying a strong electric field across the material).^[^ ^14^ ^]^ In a study on the electrospinning of PVDF, Lei et al.^[^ ^15^ ^]^ have demonstrated that the primary driver behind the formation of this ferroelectric β phase in electrospun fibers is actually the mechanical stretching experienced by the jet, rather than the poling action of the electric field. They explain that it is crucial to ensure that during whipping, most of the jet is semi‐solid so that the tensile forces can actually stretch the polymer chains. If the jet still has too much solvent, it will become thinner, leading to lower‐diameter fibers, but that would involve solvated chains slipping past each other rather than being stretched, leading to low β phase content. The key to achieving good crystallinity is to optimize the evaporation rate of the solvent, and using a blend of N‐methyl pyrrolidone (NMP) and acetone, they observed that a ratio close to 1:1 worked well in their case, producing uniform fibers with high β content. When the solvent blend had too much of either NMP (high‐boiling solvent) or acetone (low‐boiling solvent), they observed beaded fibers with lower β content.

In this work, we focus on P(VDF‐TrFE), a copolymer of vinylidene fluoride (VDF) and trifluoroethylene (TrFE). This polymer is closely related to PVDF, and differs from it only in that a hydrogen atom is replaced by a fluorine atom in a subset of its monomeric units, which confers the advantage that P(VDF‐TrFE) readily crystallizes into its ferroelectric phase (akin to PVDF's β phase) due to steric hindrances favoring trans‐linked conformations. This is thus an important and widely studied piezoelectric polymer, and is popularly electrospun. However, as seen in **Table** **1**, most existing studies involve toxic solvents in the process, which complicates the potential deployment of this versatile material in biomedical, household and food‐related applications, even though the polymer itself is not hazardous.^[^ ^16^ ^]^ A reliable protocol for electrospinning this polymer, with a stable set of process parameters to ensure reproducibility and ease of fabrication, while using only benign solvents, is thus a pressing need in the field. Further, to truly tap into the ferroelectric and piezoelectric behavior of as‐spun fibers, they must be well‐aligned so as not to mutually cancel their dipole moments at the membrane level. Alignment may also be beneficial for other applications, e.g. specialized biomedical scaffolds.^[^ ^17^ ^]^

**Table 1:** Overview of solvents used in various studies for the electrospinning of P(VDF‐TrFE), or of PVDF where no pre‐existing studies have been found involving P(VDF‐TrFE). Health hazards and BPs are taken from the respective safety data sheets (SDSs),^[^ ^18^, ^19^, ^20^, ^21^, ^22^, ^23^, ^24^ ^]^ and HSPs are from Hansen.^[^ ^25^ ^]^ Abbreviations: DMF = dimethyl formamide, NMP = N‐methyl pyrrolidone, THF = tetrahydrofuran, MEK = methyl ethyl ketone, DMSO = dimethyl sulfoxide, MPK = methyl propyl ketone.

Solvent	P(VDF‐TrFE) studies	PVDF studies (as applicable)	Main health hazards	HSPs $\left(\delta_{D},\delta_{P},\delta_{H}\right)$ [MPa^1^ *^/^* ^2^]	BP [°C]
DMF	[4, 5, 6, 7, 8, 26]	N.A.	reproductive toxin, liver/kidney toxin, irritant	(17.4, 13.7, 11.3)	153
NMP	This study	[15, 27, 28]	reproductive toxin, irritant	(18, 12.3, 7.2)	202
Acetone	[4, 8, 26]	N.A.	irritant	(15.5, 10.4, 7)	56
THF	[29]	N.A.	carcinogen, irritant	(16.8, 5.7, 8)	65
MEK	This study, [4, 5, 6, 30, 31]	N.A.	irritant	(16, 9, 5.1)	80
DMSO	This study	[28]	–	(18.4, 16.4, 10.2)	189
MPK	This study	–	irritant	(16, 7.6, 4.7)	101

All four solvents used in this study have been observed to produce visually clear and homogeneous solutions of P(VDF‐TrFE). Comparing the HSP values across these and other solvents used in the literature for this polymer, which are listed in Table 1 and are all polar aprotic solvents, it is apparent that while the $\delta_{D}$ and $\delta_{P}$ values are relatively consistent across successful solvents, there is some leeway with $\delta_{H}$. This suggests that dispersion and polar forces play a greater role than hydrogen bonding in dissolving the polymer. This is expected since P(VDF‐TrFE) contains polar monomeric units in the chain, which can interact with the polar aprotic solvent molecules to achieve solvation. However, further chemical and computational studies would be needed to verify this conjecture and further illuminate the exact mechanism of dissolution.

We consider the following criteria for solvent selection: 1) The solvent must dissolve the polymer sufficiently well, with the caveat that the solvent that most easily dissolves it is not necessarily the best choice for electrospinning.^[^ ^11^ ^]^ 2) The BP must be neither too high nor too low. This is to avoid excess solvent droplets, leading to beads, as well as premature drying, leading to dendrite formation. This is crucial for obtaining uniform, well‐aligned fibers, and for process stability, i.e., the ability to electrospin for a long time with a fixed set of parameters without the need for adjustments (a “set and forget” recipe). The evaporation rate must be optimized to keep the fiber semi‐solid in flight, for adequate chain stretching, in light of the role of mechanical stretching of polymer chains as the dominant cause of ferroelectric phase formation.^[^ ^15^ ^]^ 3) The solvent must not pose health hazards. Exposure to toxic solvents can cause cancer, infertility, liver damage, etc. Products made with toxic solvents such as dimethyl formamide (DMF) and NMP are not suitable for food, biomedical or household use, as per various national regulatory bodies.^[^ ^32^, ^33^, ^34^, ^35^ ^]^ Medical devices developed using these will face challenges during clinical approvals and will need to be re‐engineered with alternative solvents.

In this study, we explore the electrospinning of P(VDF‐TrFE) using the solvents methyl ethyl ketone (MEK) and methyl propyl ketone (MPK), from the family of ketones, dimethyl sulfoxide (DMSO), from the family of organosulfur compounds, and N‐methyl pyrrolidone (NMP), from the family of lactams. Their molecular structures are shown in Figure S1 (Supporting Information). Of these, MEK, MPK and DMSO are benign, while NMP is toxic but popular and hence used for comparison. We have also experimented with DMSO/MEK and NMP/MEK blends, which the lever rule predicts should also dissolve P(VDF‐TrFE). We study the effect of solvent volatility (i.e., BP) on both the stability of the electrospinning process and the quality of the resultant fibers. We also present a quantitative approach to characterizing the mutual alignment of electrospun fibers and define some key quality metrics for it. Having identified the solvent that optimizes electrospinning process stability and fiber quality, we further study the effect of the process parameters on the properties of the membranes produced. An optimal solvent with an intermediate BP is crucial for successful electrospinning of P(VDF‐TrFE), and a good candidate for this, that is importantly non‐toxic and environmentally benign, has been identified in this study. This is in contrast to previous studies reporting electrospinning of P(VDF‐TrFE) where toxic solvents have been commonly employed.

## 2 Results and Discussion

Electrospinning of P(VDF‐TrFE) was conducted using various solvents/blends and different experimental parameters, as discussed in detail in the Experimental Section. **Table** **2** summarizes the particulars of the fibrous membranes produced, and lists the specimen names assigned to them, which are used as identifiers in the forthcoming subsections, where results are discussed in depth.

**Table 2:** Electrospinning process summary. Membrane names are assigned based on abbreviated solvent names, and where necessary, the spinning distance and feed rate used. For depositions that were not stable, nominal parameter values that we tried to maintain along with ranges of variation have been mentioned, as the process required continuous tweaking and could not run with a constant set of values.

Membrane name	Solvent	Concentration [% w/v]	Spinning distance (SD) [mm]	Nozzle voltage [kV]	Solution feed rate (FR) [mL h^−1^]	Process stability
Me	MEK	25	40 (30–90)	10 (5–40)	1.0 (0.5–5.0)	poor
D	DMSO	25	140	15	0.5	good
D‐Me	DMSO/MEK 2:1	16.67	120	20	2.0	intermediate
N	NMP	20	110	35	0.2	good
N‐Me	NMP/MEK 5:3	25	90	30	1.0	intermediate
Mp‐90‐1	MPK	25	90	15	1.0	good
Mp‐140‐1	MPK	25	140	15	1.0	good
Mp‐60‐1	MPK	25	60	15	1.0	good
Mp‐90‐0.5	MPK	25	90	15	0.5	good
Mp‐140‐0.5	MPK	25	140	15	0.5	good
Mp‐60‐0.5	MPK	25	60	15	0.5	good

### 2.1 Choice of Solvent Affects Electrospinning Process Stability and Fiber Quality

#### 2.1.1 Process Stability and Fiber Morphology

**Figure 1:**
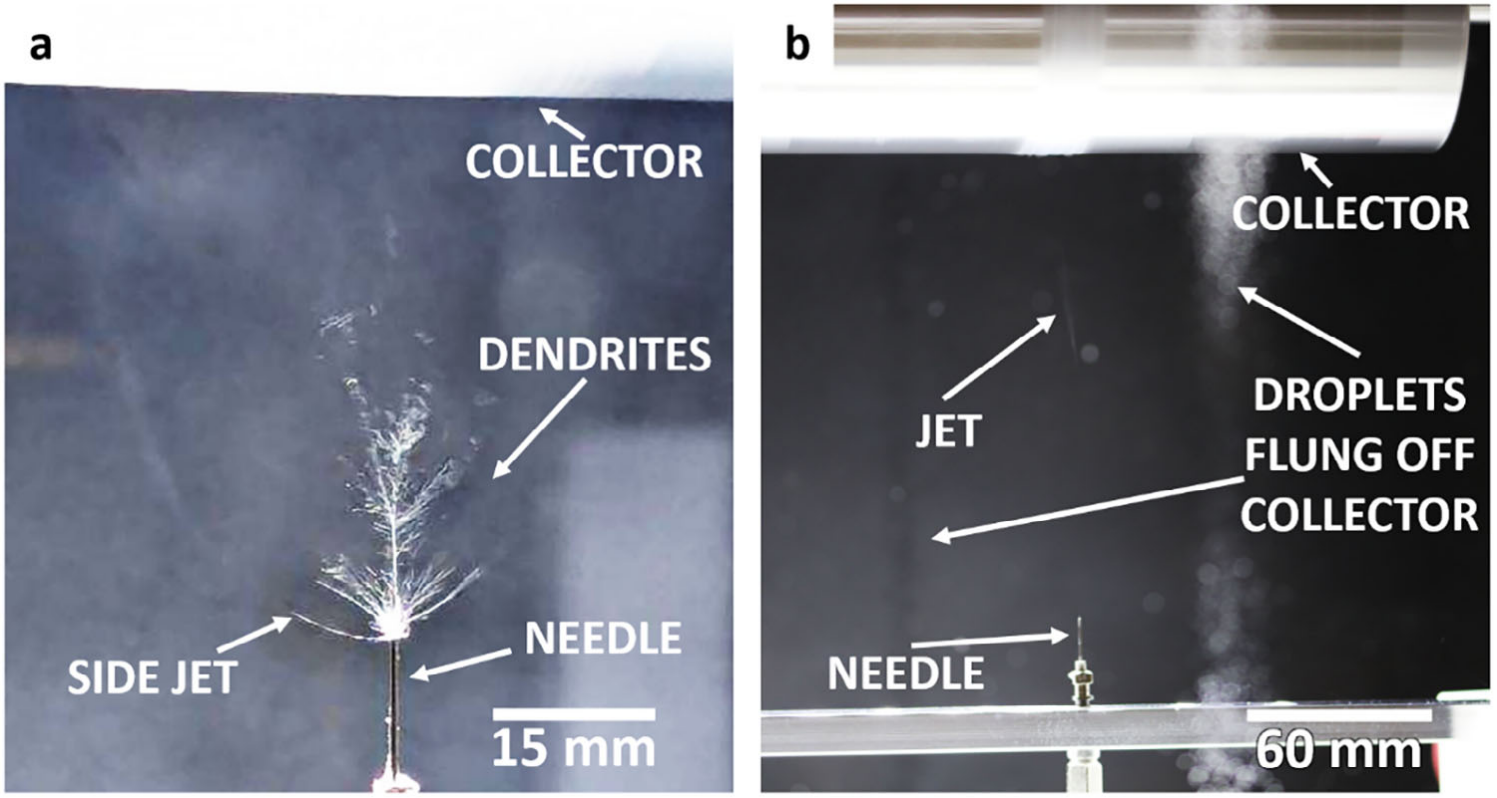
Visual experimental observations of a) dendrite formation with low‐boiling solvents, and b) excess solvent droplets with high‐boiling solvents, during electrospinning.

**Figure 2:**
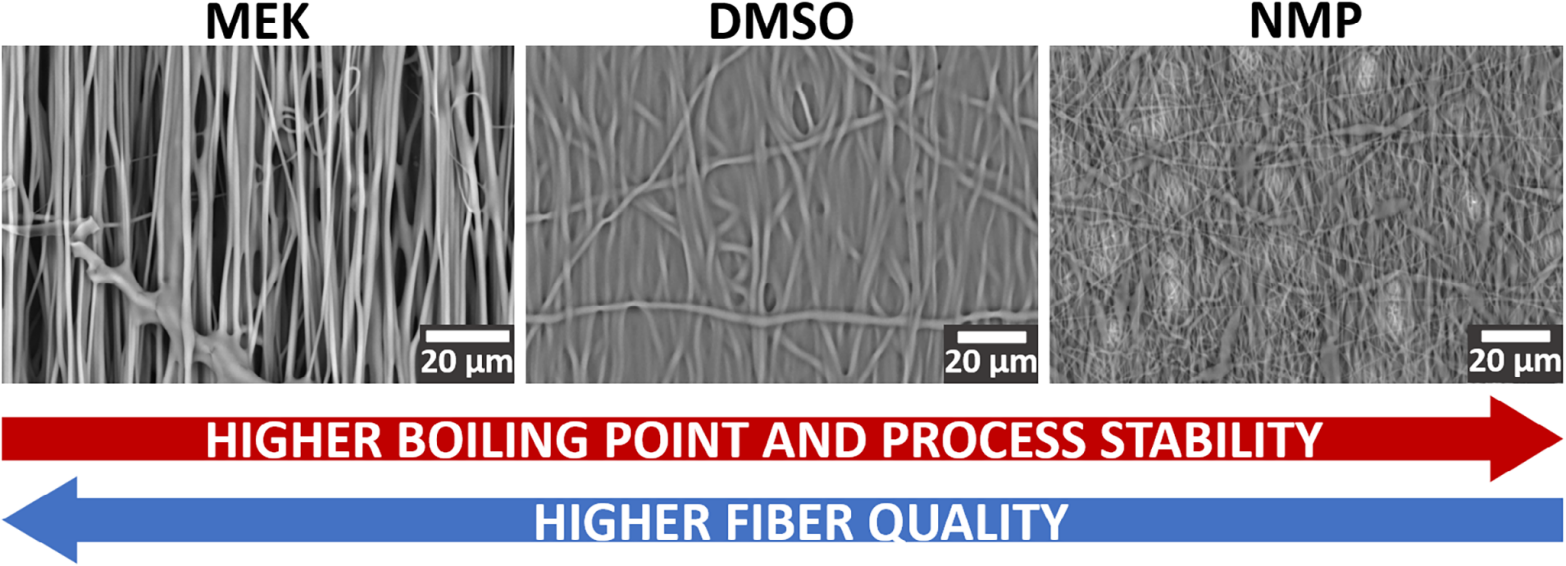
SEM images of fibers spun using low‐ and high‐boiling solvents. MEK produced uniform and well‐aligned fibers. Other solvents produced lower quality membranes with imperfections such as indistinct/welded fibers and spindle‐shaped beads.

Electrospinning with the low‐boiling solvent MEK resulted in a strong tendency for the solution jet to prematurely dry up at the tip of the needle and form dendrites of the polymer, as illustrated in **Figure** **1**. Although MEK produced well‐defined, straight and aligned fibers, as shown in the scanning electron microscopy (SEM) image in **Figure** **2**, this advantage was undermined by the needle being blocked every 1–5 min, necessitating a pause‐clean‐resume step. Upon resumption, some tweaking of the flow rate, spinning distance and nozzle voltage was necessary to successfully start deposition again, after which we could gradually return to the chosen parameter values, by which time significant dendrite formation would again take place, prompting us to repeat the previous steps. Thus, low‐boiling solvents cannot allow for a simple and consistent electrospinning recipe with a repeatable set of experimental parameters.

On the other hand, the high‐boiling solvents DMSO and NMP did not suffer from the problem of dendrite formation, and allowed the process to run smoothly with a constant set of parameter values. However, the presence of excess solvent droplets shown in the photograph of the setup (Figure 1) indicates that the fibers were still “wet” upon reaching the collector, which could promote inter‐fiber welding, de‐straightening and de‐alignment. SEM images (Figure 2) support this hypothesis.

When attempted with the mixed solvent systems of DMSO/MEK and NMP/MEK, the electrospinning process showed marginally improved stability over the case of the pure low‐boiling solvent, as the pause‐clean‐resume routine was required less frequently and parameters did not require such wide‐ranged adjustment. Although the tendency for dendrite formation was decreased, droplet formation was still observed. However, as seen in the SEM images (Figure S2, Supporting Information), there was no improvement in fiber morphology when compared to the pure high‐boiling solvents, as the membranes still had indistinct/welded fibers and patchy regions.

**Figure 3:**
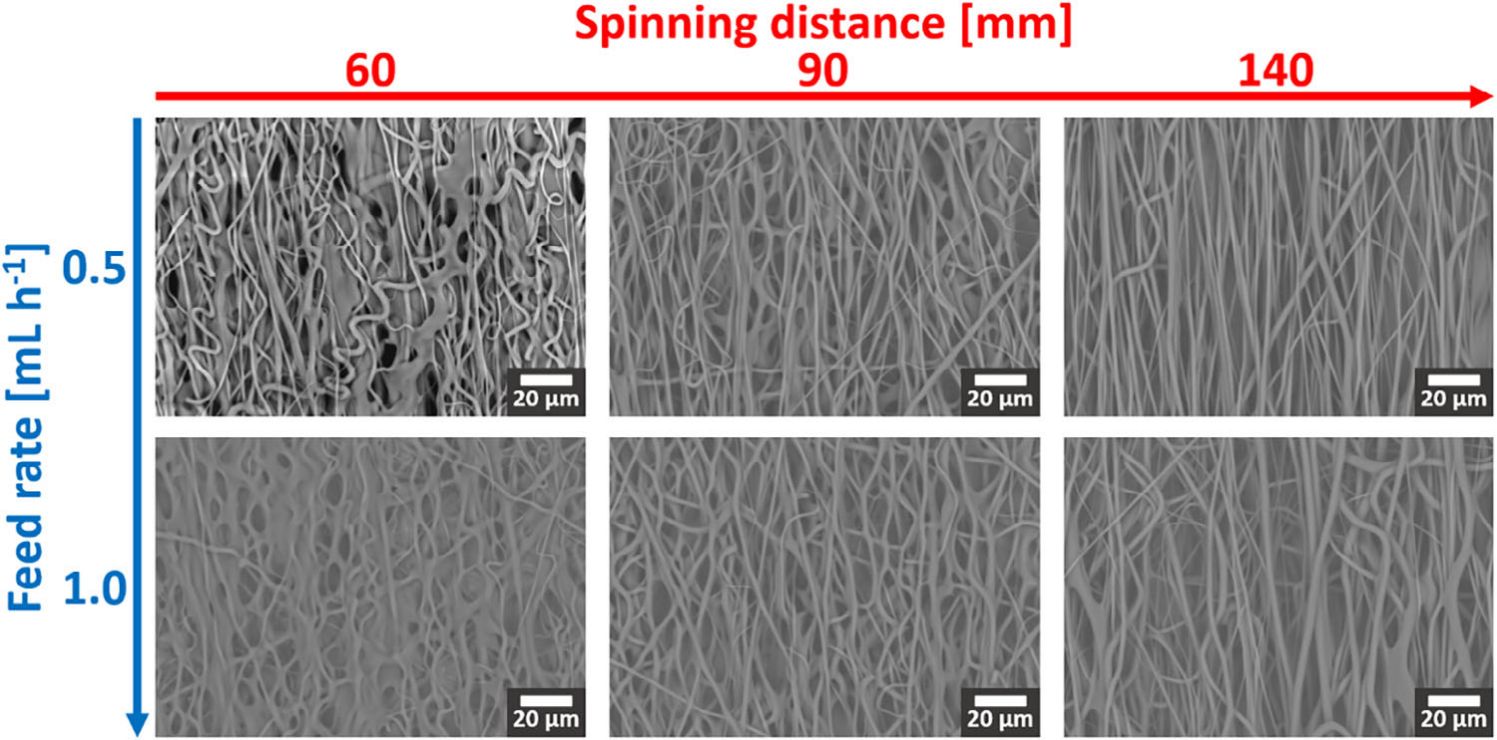
SEM images to illustrate the variation of MPK‐based electrospun fiber morphology with feed rate and spinning distance.

Finally, when electrospinning was conducted with MPK, which is a solvent of intermediate BP, we observed that the process was stable, while producing well‐defined fibers almost as well‐aligned as those produced using MEK, after some optimization of feed rate and spinning distance, which are two important factors to control the deposition. This is illustrated in **Figure** **3** and discussed in more detail later.

#### 2.1.2 Ferroelectric Phase Content

The fourier transform infrared (FTIR) spectra of the membranes produced allow estimation and comparison of their ferroelectric phase content. As discussed in our group's previous work,^[^ ^36^, ^37^ ^]^ two absorbances are considered: $f_{840}$, the intensity of the peak at ≈840 cm^−1^, and $f_{880}$, the intensity of the peak at ≈880 cm^−1^. The ratio $f_{840}/f_{880}$ is taken as an indication of ferroelectric phase content. Although this ratio is not necessarily restricted between 0 and 1 and is not a value for the phase fraction, it allows us to compare and rank similarly prepared samples. The reasoning behind considering these values is that ≈840 cm^−1^ is preferentially absorbed through symmetric stretching by CF_2_ and CC bonds belonging to chains with 3 or more trans links placed together, while ≈880 cm^−1^ is absorbed through the rocking of CF_2_ and CH_2_ and antisymmetric stretching of CF_2_, whether the trans links are scattered individually or organized together.^[^ ^38^, ^39^, ^40^, ^41^, ^42^, ^43^ ^]^

Several studies^[^ ^8^, ^26^ ^]^ have used another approach for quantifying the ferroelectric phase of P(VDF‐TrFE), which involves use of the well‐known formula for the estimated β phase content of a PVDF specimen, given in Equation 2. Such a calculation can yield a “phase fraction”, but this particular approach must be used with caution, only as a heuristic, because this formula is meant for PVDF, and P(VDF‐TrFE) does not show a distinct absorbance peak near 763 cm^−1^.

(2) $$f_{\beta }\;\left(PVDF\right)=\frac{A_{\beta }}{A_{\alpha }\frac{K_{\beta }}{K_{\alpha }}+A_{\beta }}\;$$

where $A_{\alpha }$ and $A_{\beta }$ are the characteristic absorbances of the α and β phases respectively, situated at ≈763 and ≈840 cm^−1^, and *K_α_* = 6.1 × 10^4^ cm^2^ mol^−1^ and *K_β_* = 7.7 × 10^4^ cm^2^ mol^−1^ are the respective absorption coefficients.

**Figure 4:**
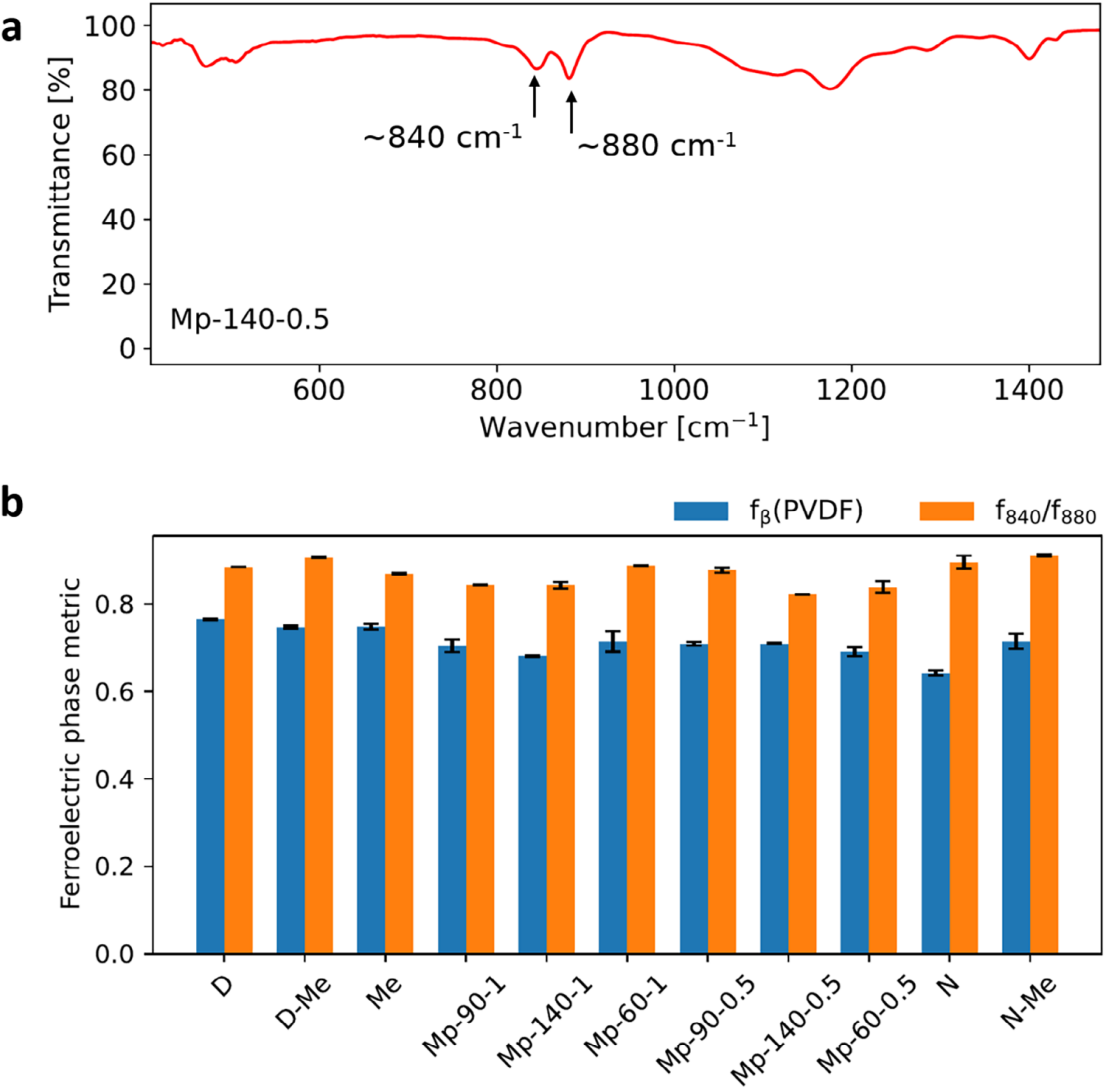
a) An example FTIR spectrum of a membrane electrospun using MPK (spinning distance: 140 mm, feed rate: 0.5 mL h^−1^). Spectra of all membranes are presented in Figures S6 and S7 (Supporting Information). b) Comparison of ferroelectric phase content of all electrospun membranes through FTIR. Measurements were taken on the top and bottom side of each specimen. Bar heights represent mean values and error bars represent standard deviations. Entries are labeled by their specimen name, as defined in Table 2.

The metrics $f_{840}/f_{880}$ and $f_{\beta }\left(PVDF\right)$, calculated for each specimen, are presented and compared in **Figure** **4**. While there is some variation visible, this is minimal in relative terms. This shows that MPK can allow for the production of electrospun P(VDF‐TrFE) fibers of ferroelectric phase content similar to that produced by other solvents, while providing an optimal combination of process stability and morphological quality.

### 2.2 Effect of Electrospinning Process Parameters when Working with MPK as a Solvent

#### 2.2.1 Fiber Alignment and Diameter

**Figure 5:**
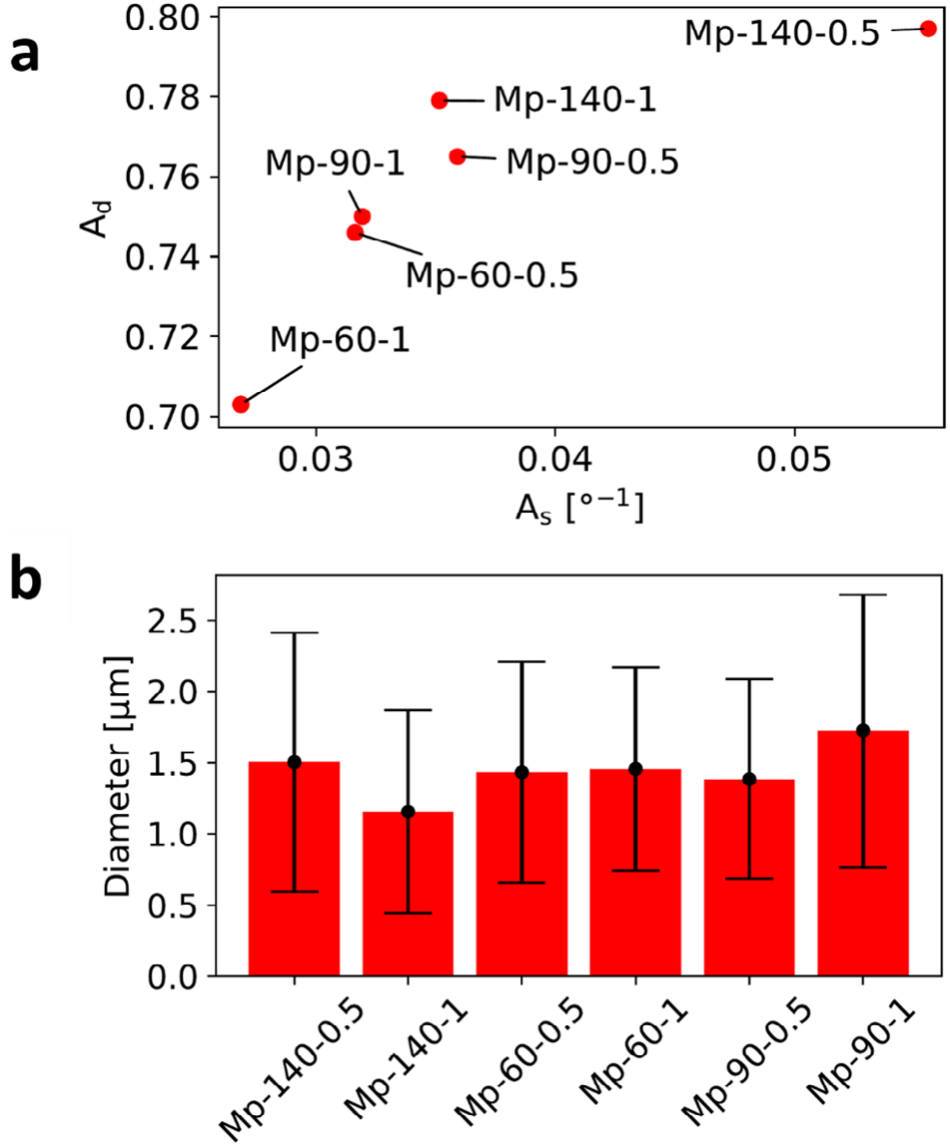
Morphological measurements on electrospun membranes produced using MPK. Data labels are specimen names, defined in Table 2. a) Alignment quality metrics (degree $A_{d}$ and sharpness $A_{s}$). b) Fiber diameter. Bar heights represent means and error bars represent standard deviations. A lower bound of 0.3 µm and an upper bound of 5.0 µm was chosen to filter out the minority of measurements with abnormally large or small values. Upon manual examination of the SEM images, it is seen that the fibers tend to lie within this range of diameter, and that any spurious measurements are due to shortcomings in the image processing, which is a function of the robustness of the measurement algorithm and the quality of image ingested. ≈70% of all the automated measurements lay within this range.

Two figures of merit, “sharpness of alignment” $A_{s}$ and “degree of alignment” $A_{d}$, have been defined in the Experimental Section to quantify the quality of alignment of fibers in an electrospun membrane. Briefly, these capture respectively the statistical sharpness (i.e., inverse spread) and degree of similarity of the orientations of numerous fiber segments. A clear trend is visible in **Figure** **5**a, which shows the dependence of these metrics on electrospinning parameters. $A_{s}$ and $A_{d}$ both increase as the spinning distance increases, keeping the feed rate fixed, and as the feed rate decreases, keeping spinning distance fixed. Overall, the specimen with the largest spinning distance and the lowest feed rate (here, ‘Mp‐140‐0.5’) shows the best alignment. This can also be qualitatively assessed from Figure 3. On the other hand, as seen in Figure 5b, the diameter of the electrospun fibers does not depend as markedly as the quality of alignment does on the process parameters. Histograms of the diameter and orientation distributions of each membrane, along with the individual measurements annotated on the SEM images, are presented in the Supporting Information (Figures S3 and S4, Supporting Information).

#### 2.2.2 Ferroelectric Phase Content

**Figure 6:**
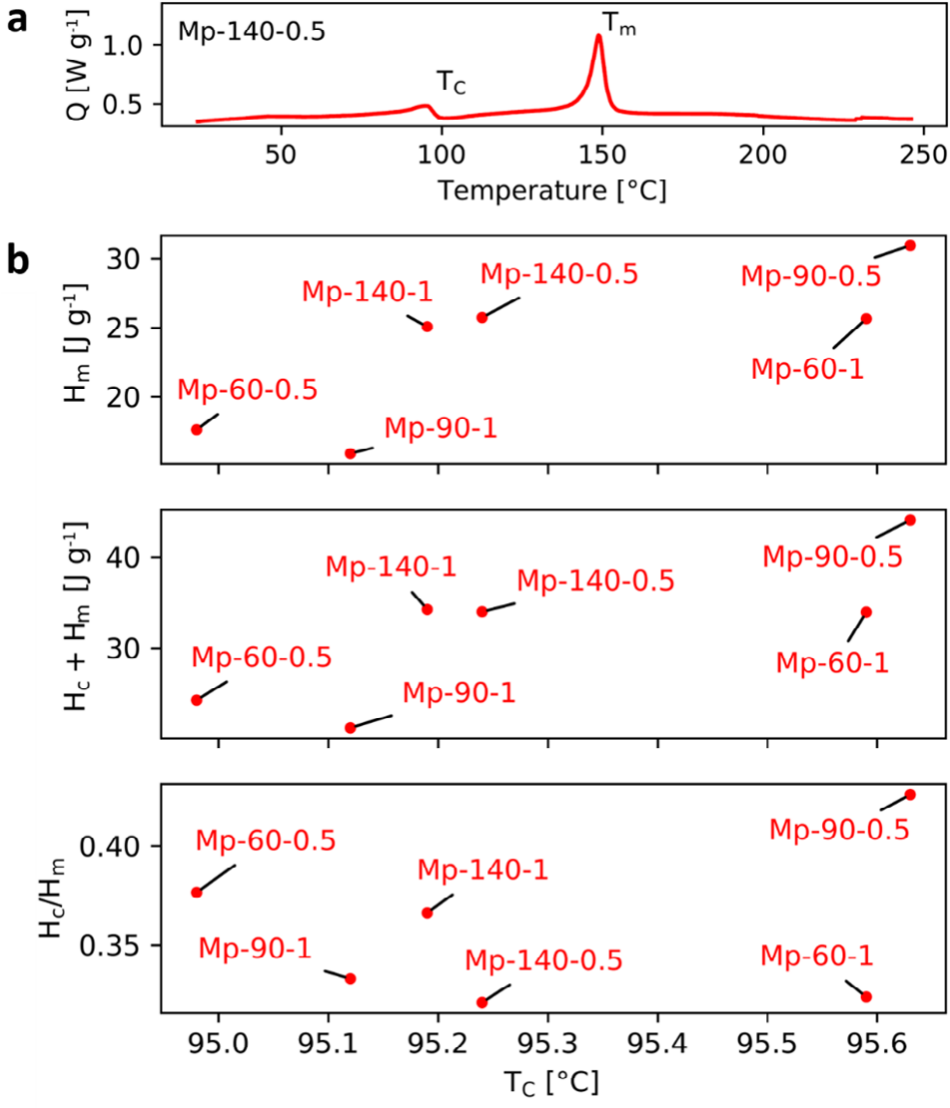
a) An example DSC thermogram of a membrane electrospun using MPK (spinning distance: 140 mm, feed rate: 0.5 mL h^−1^). *Q* is the normalized heat flow in the ‘exo down’ convention. *T*_C_ and *T*_m_ mark the Curie and melting transitions, respectively. b) Comparison of DSC ferroelectric phase content metrics against Curie temperature for MPK‐based specimens.

The differential scanning thermograms recorded for all specimens prepared using MPK are shown in the Supporting Information (Figure S8, Supporting Information) and a representative plot is presented in **Figure** **6**a. In the heating curve, we first see a peak near 95 °C that corresponds to ferroelectric‐to‐paraelectric (Curie) phase transition. Next, we see a peak near 150 °C, corresponding to melting.^[^ ^36^, ^44^, ^45^, ^46^ ^]^ Enthalpies of the ferroelectric‐to‐paraelectric phase transition ($H_{c}$) and melting ($H_{m}$) were calculated from each thermogram. For PVDF, which masks its Curie transition behind its melting transition, the degree of crystallinity is often calculated by comparing the observed and ideal (fully crystalline) melting enthalpies. However, P(VDF‐TrFE) presents a challenge here since the ferroelectric phase, which is the phase of interest, is lost long before melting. For this material, $H_{c}$ and $H_{m}$ must both be considered, and even then, an absolute value for the ferroelectric phase fraction is difficult to establish, but by comparing $H_{m}$, $H_{c}+H_{m}$ and $H_{c}/H_{m}$, we can achieve relative estimates across specimens.^[^ ^36^, ^37^, ^47^, ^48^ ^]^ These metrics have been plotted against the Curie transition temperature ($T_{C}$) and compared across the specimens in Figure 6b. Overall, the ‘Mp‐90‐0.5’ sample ranks the highest in all metrics and has the highest transition temperature, indicating the highest amount of ferroelectric phase. This can be understood by considering the effects of solution feed rate and spinning distance individually. The lower feed rate of 0.5 mL h^−1^ encourages more stretching of the jet by supplying less solution at any given time, and hence specimens prepared with this setting generally tend to be more crystalline (as indicated by higher $H_{m}$). The effect of spinning distance is more nuanced: A value of 90 mm results in a stronger electric field than 140 mm for the same voltage, meaning there would be stronger stretching. However, at 60 mm, even with a stronger electric field, there is generally lower crystallinity. We believe that this is because at such short distances and high accelerations, there is not enough flight time available and the jet reaches the collector before completely drying up and using its full potential to stretch. This is consistent with the patchy regions and curled fibers seen in the SEM images of the 60 mm specimens.

**Figure 7:**
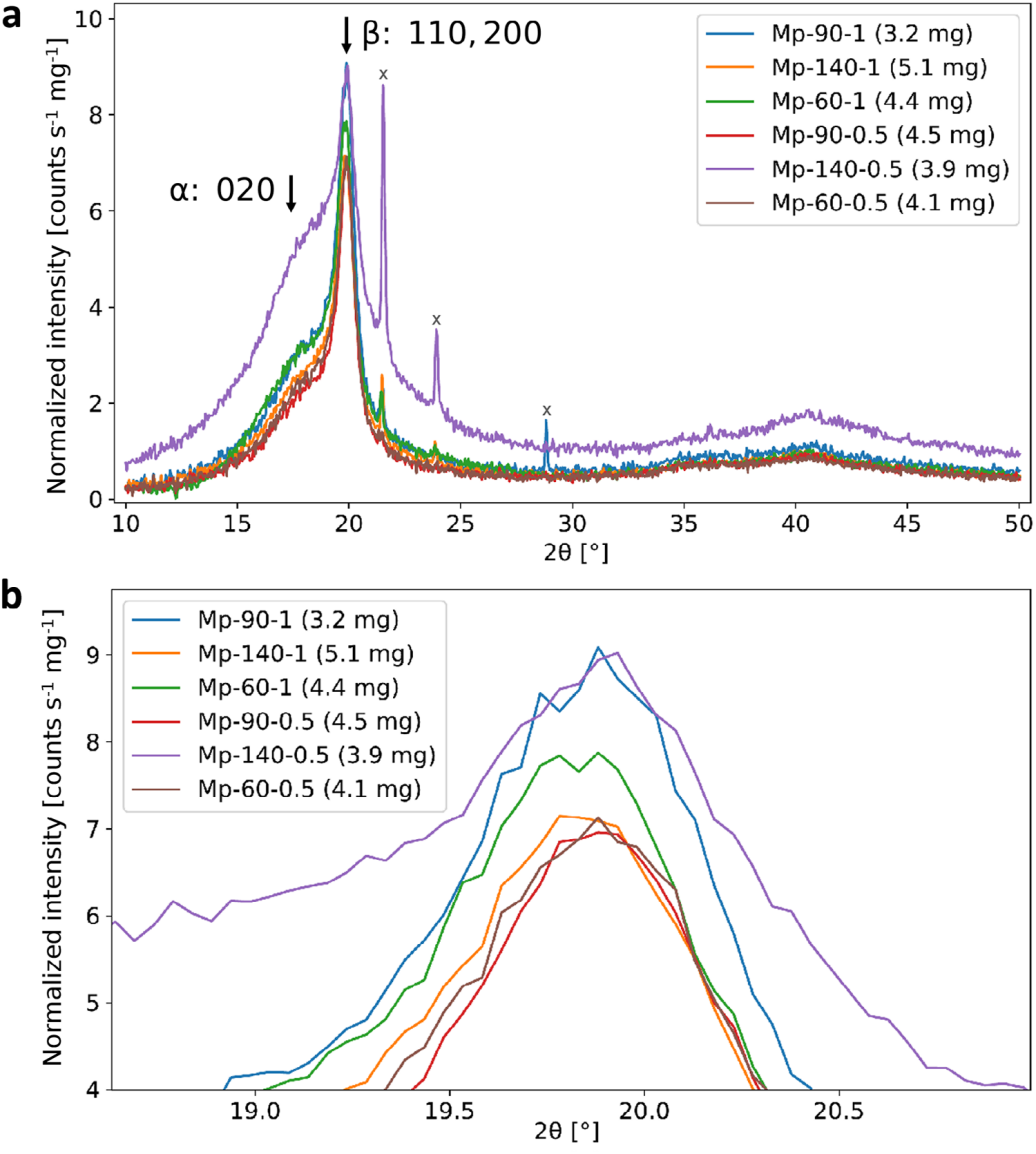
Normalized XRD spectra of the MPK‐processed membranes: a) overall view and b) zoomed in on the principal peaks. Out of the spurious peaks (marked ‘x’), the smaller, sharp peak at ≈28° has been assigned to Si (111), probably caused by a scratch on the low‐background Si wafer. Those at ≈21° and ≈24° have been assigned to polyethylene, a very common contaminant during sample handling and a ubiquitous polymer that has two sharp peaks at these locations (see Figure S9, Supporting Information for details).

**Figure** **7** shows the X‐ray diffractograms produced by the six MPK‐processed membranes. The large peak at ≈19.9°, present in all specimens, corresponds to the (110) and (200) planes of the ferroelectric phase.^[^ ^49^, ^50^ ^]^ A shoulder near 17.5°, associated with the (020) planes of the paraelectric phase,^[^ ^49^, ^50^ ^]^ is also present. This is most prominent in the ‘Mp‐140‐0.5’ specimen, which also has an overall elevated noise floor, which may have been due to the relatively larger amount of Vaseline that was required to hold it down. In a follow up study, we intend to develop a more detailed method to quantify the ferroelectric (crystalline) phase content of electrospun P(VDF‐TrFE) fibers using XRD.

## 3 Conclusion

This study presents a facile method to produce P(VDF‐TrFE) fibers through electrospinning, by introducing MPK as a new solvent for this technique. The high level of process stability enabled by this solvent affords a remarkable ease and consistency in fabrication endeavors to produce high‐quality fibrous membranes. This is all achieved while minimizing health and environmental risks due to the non‐toxic nature of the solvent used.

The reason why MPK works well for electrospinning P(VDF‐TrFE) may be understood by comparing its chemical nature to MEK, which is a solvent established to some extent in existing studies that involve solutions of P(VDF‐TrFE). These are both unbranched aliphatic ketones with the keto group in the same position (C_2_) (see Figure S1, Supporting Information), and have similar properties as solvents, as is apparent from their similar HSP values in Table 1. However, the slightly longer chain of MPK results in a higher BP, which is beneficial from the perspective of process stability. The optimal BP in our case has been found to be that of MPK (≈100 °C), but other laboratory environments and experimental setups may necessitate a different BP. Equally, one may wish to electrospin a different polymer that one knows dissolves in a different type of solvent, but which happens to have an unsuitable BP. For such cases, the logic of this study may be extended by choosing another solvent, of a similar chemical nature and nearby HSP values to a known performer, but with a subtle difference that primarily impacts physical properties (such as BP) rather than chemical properties that may affect its solvency. One way approach to this could be to increase the chain length in a region of the solvent molecule away from any functional groups. For example, if the polymer to electrospin is still P(VDF‐TrFE) but a further jump in BP is required, an even longer ketone of a very similar structure, such as 2‐heptanone, may be tried. We envisage that this framework for optimizing solvency and BP will be of use to researchers attempting the development of new electrospinning protocols.

Additionally, we have investigated the impact of electrospinning process parameters on fibers produced from a P(VDF‐TrFE)/MPK solution. In the electrospinning chamber, the electric field (which decreases with spinning distance and increases with nozzle voltage) determines the stretching forces acting on the leading end of the jet. At the same time, the flow rate determines how much solution is made available at the trailing end of the jet to replace the material propelled away by the field. Additionally, the spinning distance also affects the flight time available to the jet, which determines how much of the jet's potential to stretch is realized before it solidifies or reaches the collector. Thus, the degree of stretching of the jet has a direct relationship with the nozzle voltage, an inverse relationship with the flow rate, and a nuanced relationship with the spinning distance (an inverse factor through the electric field and a limited direct factor through the flight time). Now, if the jet is relatively wet due to a large amount of unevaporated solvent remaining, the jet elongation observed may happen as a result of the solvated chains slipping past each other, but if the amount of solvent is low due to adequate evaporation and the jet is already semi‐solid, the stretching force acts on the individual polymer chains themselves. In this scenario, chains that are in the trans‐gauche‐trans‐gauche’ conformation (corresponding to the undesirable α phase) get transformed into the all‐trans conformation (corresponding to the desirable β phase), which is the most stretched‐out chain conformation for this polymer.^[^ ^15^ ^]^ Further, we have observed through quantitative assessments that the quality of mutual alignment of the electrospun fibers has an inverse relationship with the feed rate and a direct relationship with the spinning distance. We hope that our approach to characterizing fiber alignment through the quality metrics $A_{s}$ and $A_{d}$, and the insights on the effects of process parameters presented in this paper, will be of help to the electrospinning community.

## 4 Experimental Section

### Materials

Poly(vinylidene fluoride‐co‐trifluoroethylene) (Piezotech FC30, 70:30 molar P(VDF‐TrFE) powder), with a nominal molecular weight of 450 kg mol^−1^ was purchased from Arkema, France. Dimethyl sulfoxide (DMSO) was purchased from Honeywell. Methyl propyl ketone (MPK) (also known as 2‐pentanone) and methyl ethyl ketone (MEK) (also known as 2‐butanone) were purchased from Thermo Scientific. N‐methyl pyrrolidone (NMP) was purchased from Sigma–Aldrich. All substances were used as purchased without further purification.

### Polymer Solution Preparation

Various solutions of P(VDF‐TrFE) in the solvents DMSO, DMSO/MEK 2:1 by volume, MEK, MPK, NMP, and NMP/MEK 5:3 by volume were prepared at concentrations of 25%, 16.67%, 25%, 25%, 20%, and 25% w/v, respectively. This was done by mixing the polymer and each solvent/blend in a closed glass vial on a laboratory magnetic stirrer equipped with a hot plate, running at 60–240 RPM and 55–90 °C for 1–3 days, to get visually clear and homogeneous solutions of workable viscosities.

### Electrospinning

Membranes of P(VDF‐TrFE) fibers were produced using an electrospinner (NS24, Inovenso, Turkey). Solvent electrospinning with a rotating drum as collector was the method of choice as the applied strong electric field would promote in situ poling of the polymer chains within each fiber,^[^ ^51^ ^]^ and the rotating drum would favor mutual alignment of the fibers in the membrane, which would ultimately result in a consistent overall alignment of dipoles within the polymer membrane, an important requirement for uncovering the piezoelectric behavior of the material. Standard electrospinning practice involves first feeding the solution into the nozzle, obtaining a solution droplet at the tip, and then turning on the electric field to start the deposition.^[^ ^52^ ^]^ However, in the experiments conducted in this work, the rest of the process (drum rotation, electric field, etc.) was started first, and the solution was then introduced into the tip as the last step, as that was found to improve process stability, with a lesser tendency to form multiple jets and nozzle‐blocking dendrites. This is because having a round droplet at the tip exposes a lot of solution to the atmosphere and the electric field, allowing for the rapid expulsion of multiple jets and the formation of dendrites.

All depositions were carried out at room temperature (20 °C). The nozzle used was a 21‐gauge needle supplied by the electrospinner manufacturer, and the collector was aluminum foil, wrapped on a conductive drum of diameter 10 cm, rotating at 2000 RPM. With the electrospinner casing grounded (0 V), the collector was held at a voltage of ‐10 kV for preferential deposition on the substrate, and the nozzle was held at various positive voltages. The process parameters used for each membrane produced are specified in Table 2.

### Scanning Electron Microscopy (SEM) and Morphology Analysis

A scanning electron microscope (SEM) (TM3030Plus, Hitachi, Japan) was used to image the electrospun polymer membranes. The backscattered electron (BSE) signal was used, at an accelerating voltage of 15 kV, and a working distance of 4–7 mm. The micrographs taken at 1000x magnification were then analyzed using custom proprietary image processing code, to measure diameters and orientation angles at 1000 randomly selected points along the fibers.

The distribution of fiber orientations can cast light on how well‐aligned the electrospun fibers are. Accordingly, two metrics to characterize the quality of alignment are defined as follows:

- Sharpness of alignment ($A_{s}$):
(3) $$A_{s}=\frac{1}{\sigma }$$

- Degree of alignment ($A_{d}$):
(4) $$A_{d}=\frac{N_{S}}{N}$$

where, σ is the standard deviation of the measured orientation angles, $N_{S}$ is the number of measurments within 1 standard deviation of the mean orientation angle, and N is the total number of measurments.

$A_{s}$ quantifies the sharpness of the orientation distribution, with a high $A_{s}$ indicating more consistently oriented fiber segments. On the other hand, $A_{d}$ measures the concentration of the measured orientation angles around the mean, relative to the spread defined by the standard deviation (or equivalently, the sharpness indicated by $A_{s}$). $A_{d}$ describes how “typical” the mean value is within the observed data. This quantity is equivalent to the “Amount” output of the Directionality plugin in ImageJ^[^ ^53^ ^]^ that specifies the fraction of observations within one standard deviation of the dominant orientation angle. A high $A_{d}$ suggests that more of the observed angles are clustered closely around the mean, while a low $A_{d}$ may suggest the presence of outliers, multiple modes, or inherent variability in the data. Moreover, $A_{d}$ depends on the type of statistical distribution. As illustrative examples, if the ideal uniform, Gaussian, and Laplacian distributions are considered, the expected values for $A_{d}$ can be calculated to be ≈0.58, ≈0.68, and ≈0.76 respectively, independent of the specific parameters (mean, standard deviation, etc.) of each distribution (please see Table S2 and Figure S5, Supporting Information for details). For other more complex distributions, $A_{d}$ may depend on the distribution parameters instead of being constant. If the type of distribution followed by the data, or a specific relationship between $A_{d}$ and the standard deviation is known, then just a single figure of merit (namely, $A_{s}$) would be sufficient to describe the quality of alignment of the fibres. However, since the type of statistical distribution applicable to the data is not known in general, both $A_{s}$ and $A_{d}$ must be investigated and may provide a more comprehensive picture than any single figure of merit.

Making automated measurements of fiber orientation and applying the statistical treatment as described here confers several advantages over traditional manual and qualitative approaches. Calculating figures of merit ($A_{s}$ and $A_{d}$) for alignment based on a large statistical dataset allows for unambiguous comparison, and offers a way to rank a number of specimens in terms of their quality of fiber alignment. The same is not possible with traditional qualitative assessments, which may vary from assessor to assessor and might not adequately distinguish between many different specimens. Further, our automated technique can make a large number of measurements (of the order of a few thousand measurements) relatively quickly. Manually measured fiber orientations may indeed be analyzed through the statistical approach, but that can be slow and inefficient, and may suffer from inherent biases in fiber selection, length delimitation and angle measurement. Thus, our approach of making automated measurements and computing these statistical quality metrics from them paves the way for further automation by integrating large‐scale quality control into the fabrication process chain, without the need for a slow and inadequate manual review step.

### Fourier Transform Infrared (FTIR) Spectroscopy

An FTIR spectrometer (iS50, Thermo Fisher Scientific, Germany) was used in attenuated total reflectance (ATR) mode, with a Polaris long life He–Ne laser, a diamond crystal, and a built‐in clamping tool. Specimens were cut out of the electrospun membranes with a focus on central regions that were not damaged or touched with tweezers. Measurements were made on both the top (free) and bottom (peeled off the substrate) faces of the membrane. For each run, 8 scans were taken and averaged, and a spectrum was produced at a resolution of 4 cm^−1^, for a range of 400–4000 cm^−1^.

### Differential Scanning Calorimetry (DSC)

Specimens weighing 2–5 mg were taken from the electrospun P(VDF‐TrFE)/MPK films by removing the substrate and using parts cut out of the central region, untouched by substrate removal tools. These were then encapsulated in aluminum pans with aluminum lids that were press‐sealed (Tzero, TA Instruments, USA). The sealed sample pans were loaded into a differential scanning calorimeter (Discovery DSC 2500, TA Instruments, USA), alongside an empty reference pan and lid of the same type and very nearly the same mass. The instrument was configured to equilibrate each specimen to a chosen minimum temperature of 20 °C, and then heat it up at 10 °C min^−1^ to a chosen maximum temperature of 250 °C. Heat flux as a function of temperature was recorded and then analyzed using the manufacturer's proprietary software (TRIOS DSC, TA Instruments, USA).

### X‐Ray Diffraction (XRD)

The membranes electrospun using MPK were characterized through the method of X‐ray diffraction (XRD) to gain insight into the different phases formed in each specimen. The diffractometer (D8 Advance, Bruker, Germany) was set up with a Cu‐K_α_ source (K_α1_: 1.5406 Å, K_α2_: 1.5445 Å) for a scan range of 10° to 50° (2θ) with an increment of 0.05° and a dwell time of 1 s per step. A low‐background Si wafer was used as the sample holder, which was rotated at 60 RPM. The anti‐scatter mechanism was a metal knife edge fixed 10 mm away from the sample.

The samples were all weighed and it was ensured that all of the material was illuminated under the beam, by attaching it to a small central region of the silicon disc using Vaseline petroleum jelly. All measurements were conducted in the same session, and an experimental background was recorded using a blank sample holder. To normalize the data for analysis, this background was subtracted (to remove any environmental or instrumental effects) from each diffractogram, and the result was divided by the specimen's mass, since diffracted intensity is proportional to the number of scattering sites (atoms in crystalline unit cells, etc.) exposed to the beam.

## Conflict of Interest

The authors declare no conflict of interest.

## Supporting information



Supporting Information

## Data Availability

The data that support the findings of this study are available at the University of Cambridge Apollo data repository (DOI: https://doi.org/10.17863/CAM.116720).
