# Supplementary material for: Electrospinning of Well‐Aligned P(VDF‐TrFE) Fibers Using a Benign Solvent
Source: Macromol Rapid Commun. 2025 Mar 29;46(13):2500099. doi: 10.1002/marc.202500099 (PMC12227229; doi:10.1002/marc.202500099)
Supplement: Supplementary file 1 — Supporting Information [file MARC-46-2500099-s001.pdf]

Macromolecular Rapid Communications

# Supporting Information

for *Macromol. Rapid Commun.*, DOI 10.1002/marc.202500099

Electrospinning of Well-Aligned P(VDF-TrFE) Fibers Using a Benign Solvent

*Shaashwat Saraff, Kalyan Ghosh, Thiyagarajan Natarajan, Giulio Isacco Lampronti and Sohini Kar-Narayan**

**Supporting Information**

**Electrospinning of well-aligned P(VDF-TrFE) fibers using a benign solvent**

*Shaashwat Saraff, Kalyan Ghosh, Thiyagarajan Natarajan, Giulio Isacco Lampronti, Sohini Kar-Narayan**

S. Saraff, K. Ghosh, T. Natarajan, G.I. Lampronti, S. Kar-Narayan
Department of Materials Science and Metallurgy, University of Cambridge
E-mail: sk568@cam.ac.uk

## 1. Molecular structures and Hansen solubility parameters (HSPs)

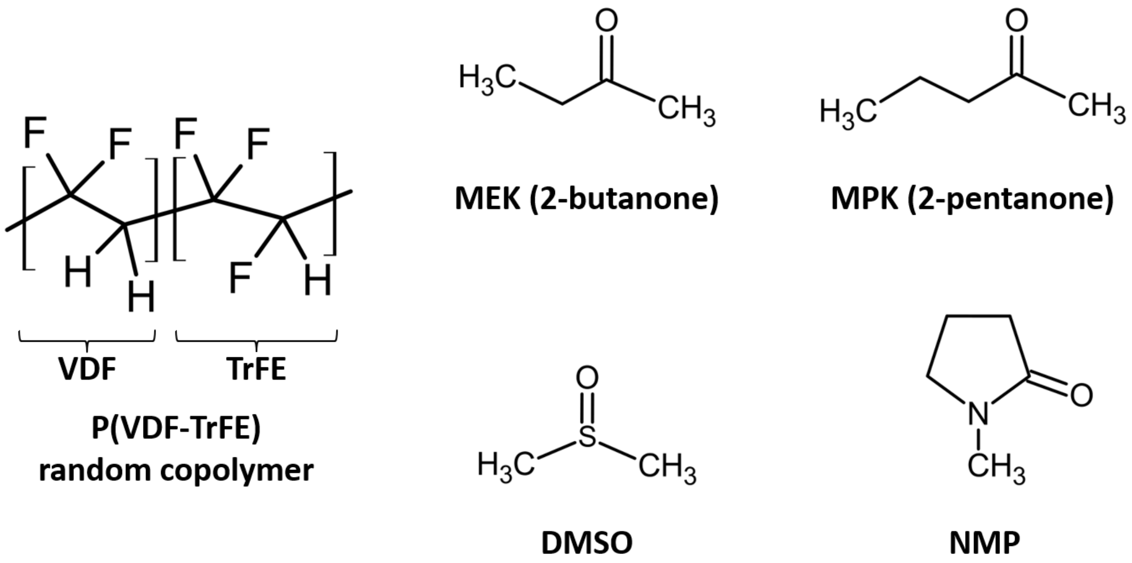


**Figure S1.** Molecular structures of the polymer and solvents used in this study

In view of the current unavailability of numerical values for the HSPs of P(VDF-TrFE) in the literature, we compare below the $D_{12}$ values between the solvents mentioned in this study and the closely related polymer PVDF, the HSPs of which have previously been determined by Bottino et al.[1] While this should offer some insight into the trend of solvent behavior, it is important to keep in mind here that since PVDF and P(VDF-TrFE) are chemically different polymers, only limited parallels may be drawn between these calculations and the results observed with P(VDF-TrFE).

**Table S1.** Comparison of Hansen distances between PVDF and the solvents discussed in this study

| Substance | $\delta_D$ [MPa$^{1/2}$] | $\delta_P$ [MPa$^{1/2}$] | $\delta_H$ [MPa$^{1/2}$] | Reference | $D_{12}$ (solvent-PVDF) [MPa$^{1/2}$] |
|---|---|---|---|---|---|
| PVDF | 17.2 | 12.5 | 9.2 | [1] | – |
| NMP | 18 | 12.3 | 7.2 | [2] | 2.569 |
| MEK | 16 | 9 | 5.1 | [2] | 5.901 |
| DMSO | 18.4 | 16.4 | 10.2 | [2] | 4.687 |
| MPK | 16 | 7.6 | 4.7 | [2] | 7.072 |
| DMF | 17.4 | 13.7 | 11.3 | [2] | 2.452 |
| Acetone | 15.5 | 10.4 | 7 | [2] | 4.562 |
| THF | 16.8 | 5.7 | 8 | [2] | 6.951 |

## 2. Scanning electron microscopy (SEM)

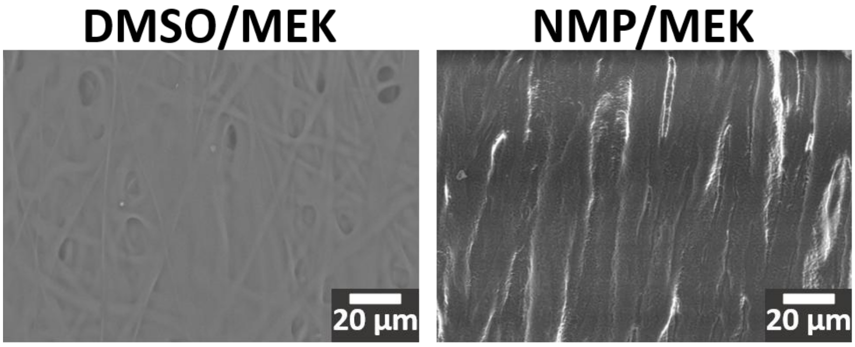


**Figure S2.** SEM images of fibers spun using blends of low- and high-boiling solvents

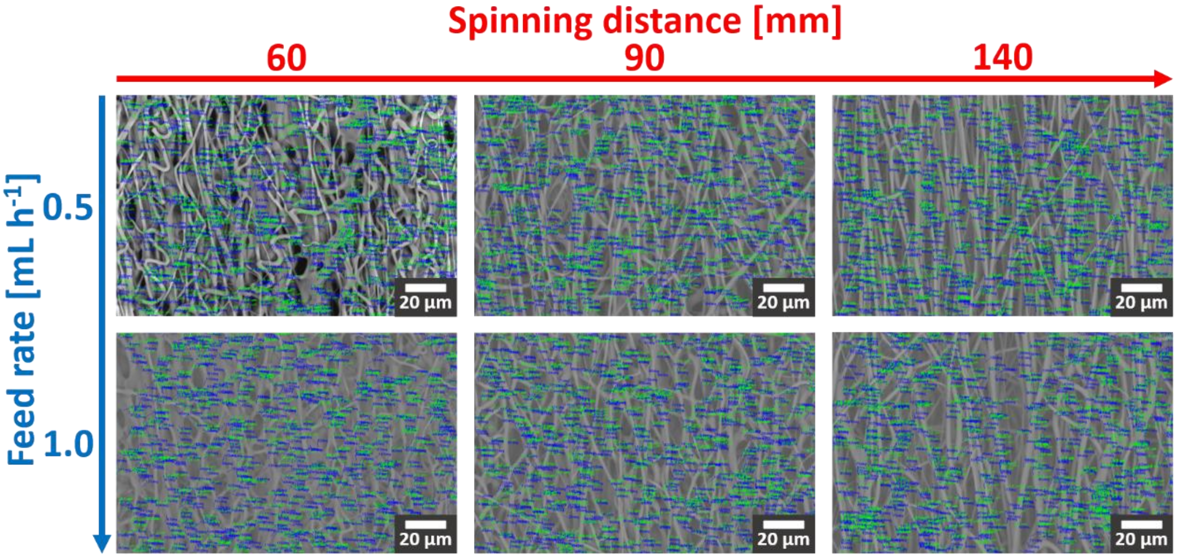


**Figure S3.** Visualization of diameter (green) and orientation (blue) measurements at 1000 randomly sampled points along fibers visible in SEM images of the MPK-processed electrospun membranes

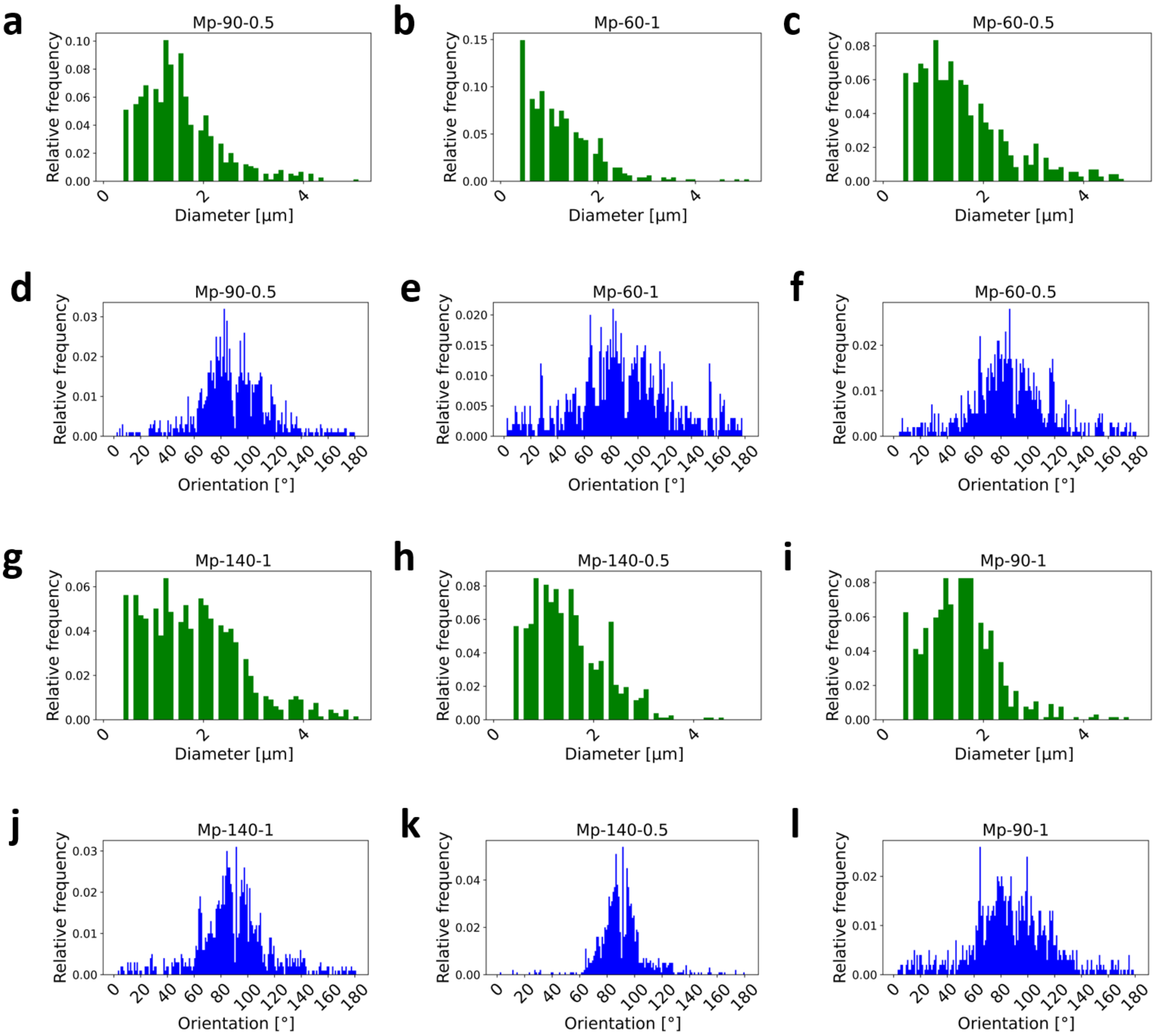


**Figure S4.** Histograms depicting diameter (a-c, g-i) and orientation (d-f, j-l) distributions calculated from the SEM images of MPK-based membranes

**Table S2.** Examples to illustrate $A_s$ and $A_d$ for different distributions. The expressions for the various quantities listed are well-known standard results which can be derived easily. For the three distributions discussed here, $A_d$ has a constant expected value since all parameters cancel out when the curve is integrated and the limits are substituted. This is not necessarily true in general, and for more complex distributions $A_d$ may depend on the parameters.

| Distribution | Normalized probability density ($p(x)$) | Mean ($x_m$) | Standard deviation ($x_s$) | $A_s = \frac{1}{x_s}$ | $A_d = \int_{x_m-x_s}^{x_m+x_s} p(x)dx$ |
|---|---|---|---|---|---|
| Uniform | $\begin{cases} p_0 \text{ if } a \le x \le b \\ 0 \text{ otherwise} \end{cases}$ where $a$ and $b$ $(a < b)$ are the bounds and $p_0 = \frac{1}{b-a}$ | $\frac{a+b}{2}$ | $\frac{b-a}{\sqrt{12}}$ | $\frac{\sqrt{12}}{b-a}$ | $\sim 0.58$ |
| Gaussian | $\frac{1}{\sigma\sqrt{2\pi}} \exp\left(-\frac{(x-\mu)^2}{2\sigma^2}\right)$ | $\mu$ | $\sigma$ | $\frac{1}{\sigma}$ | $\sim 0.68$ |
| Laplacian | $\frac{1}{2c} \exp\left(-\frac{\lvert x-\mu \rvert}{c}\right)$ | $\mu$ | $\sqrt{2}c$ | $\frac{1}{\sqrt{2}c}$ | $\sim 0.76$ |

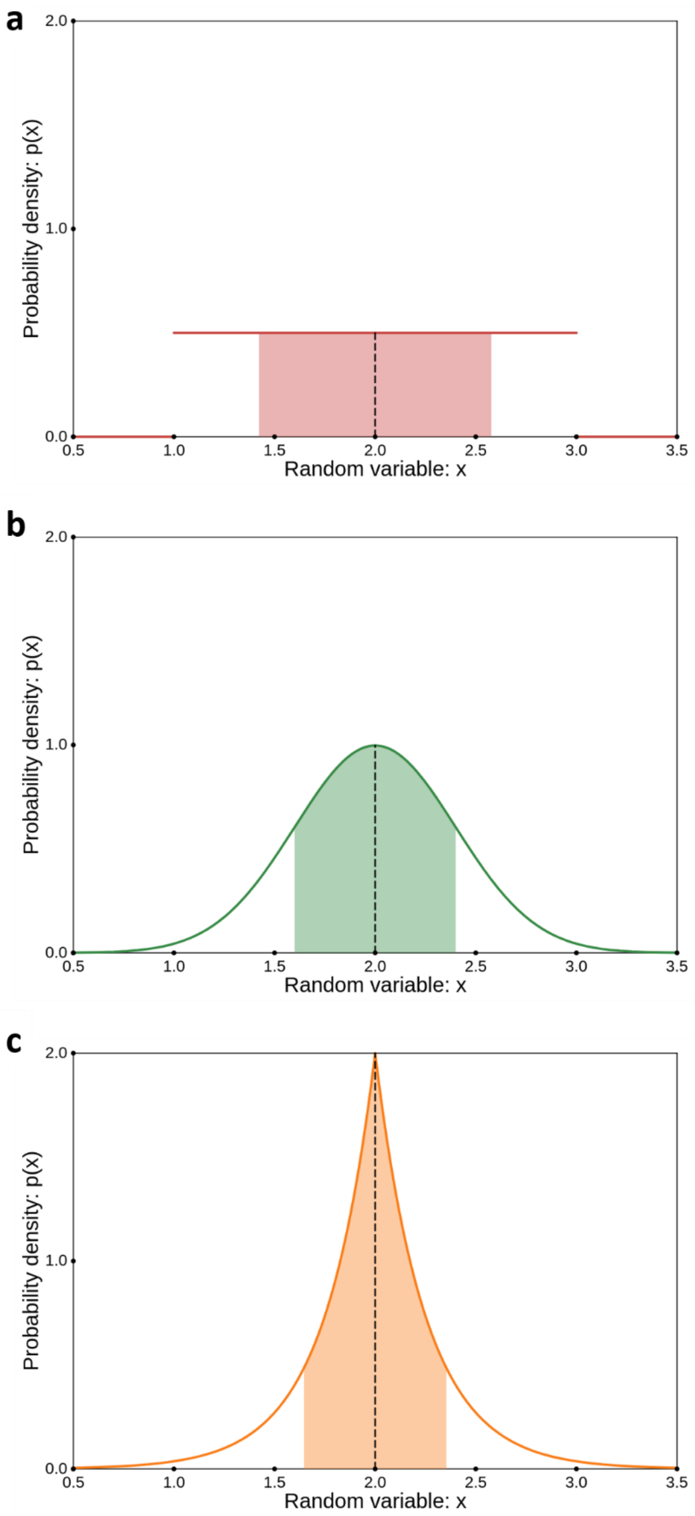


**Figure S5.** Example plots for the models discussed in **Table S1**. The mean value is annotated with a dashed vertical line and the area under the curve within one standard deviation of the mean is shaded. (a) Uniform distribution, parameters: $a = 1,\ b = 3$. (b) Gaussian (normal) distribution, parameters: $\mu = 2,\ \sigma = 0.4$. (c) Laplacian distribution, parameters: $\mu = 2,\ c = 0.25$.

## 3. Fourier transform infrared (FTIR) spectroscopy

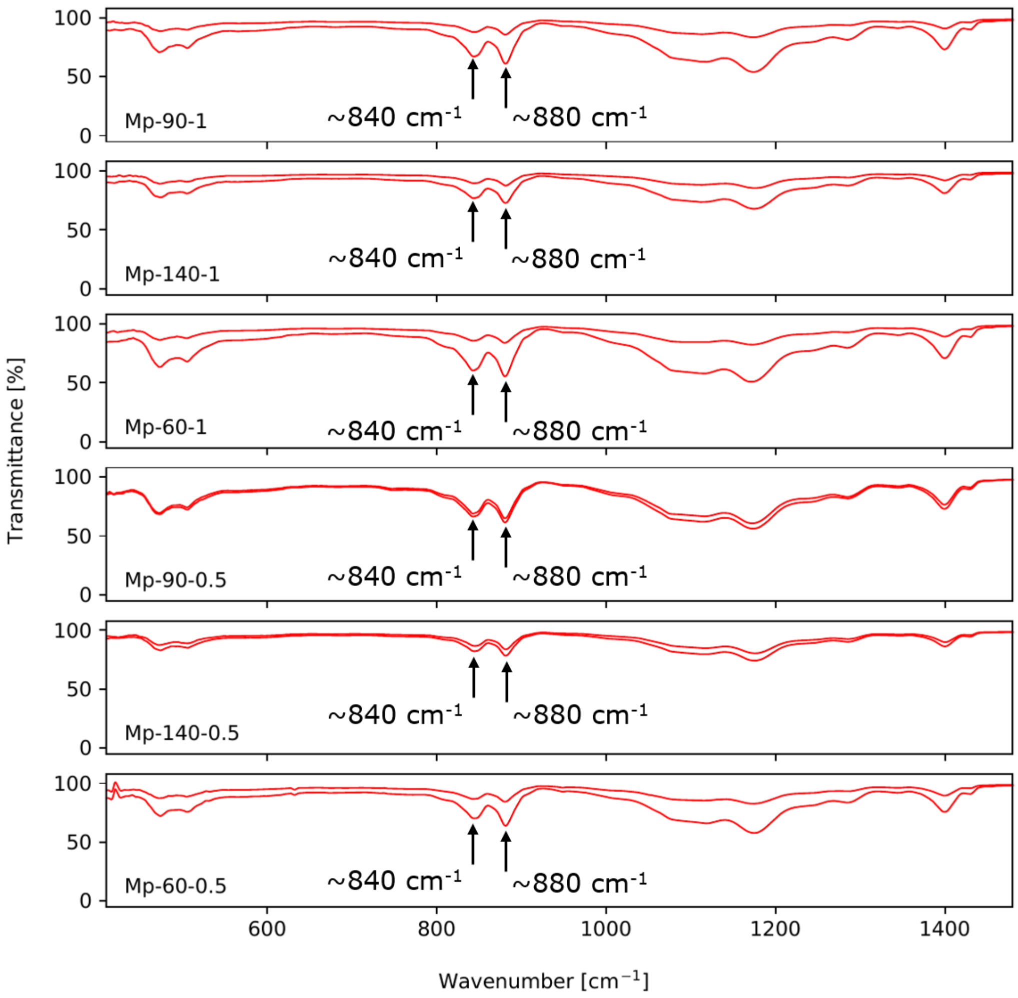


**Figure S6.** Individual FTIR transmittance spectra for MPK-based membranes. The transmittance troughs (i.e., absorbance peaks) situated at ~840 $cm^{-1}$ and ~880 $cm^{-1}$ are annotated.

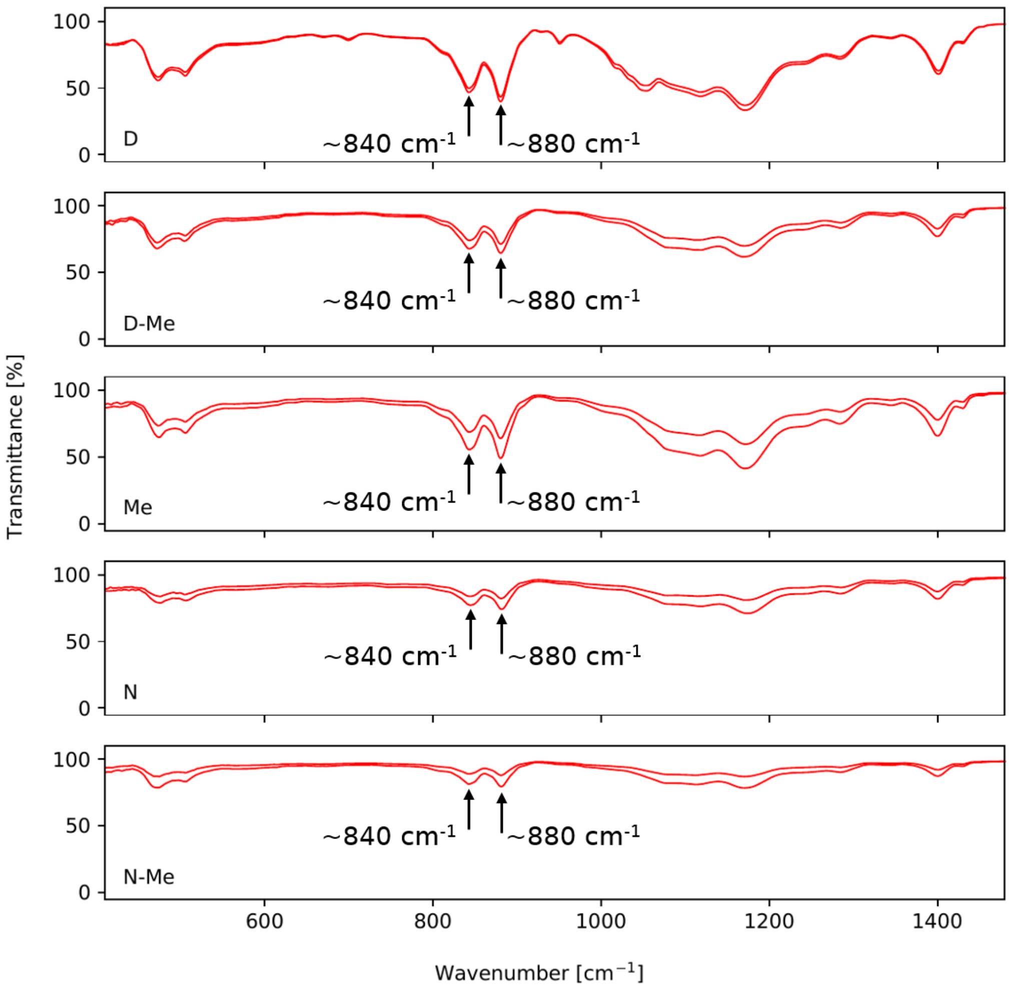


**Figure S7.** Individual FTIR transmittance spectra for membranes based on other solvents. The transmittance troughs (i.e., absorbance peaks) situated at ~840 cm$^{-1}$ and ~880 cm$^{-1}$ are annotated.

## 4. Differential scanning calorimetry (DSC)

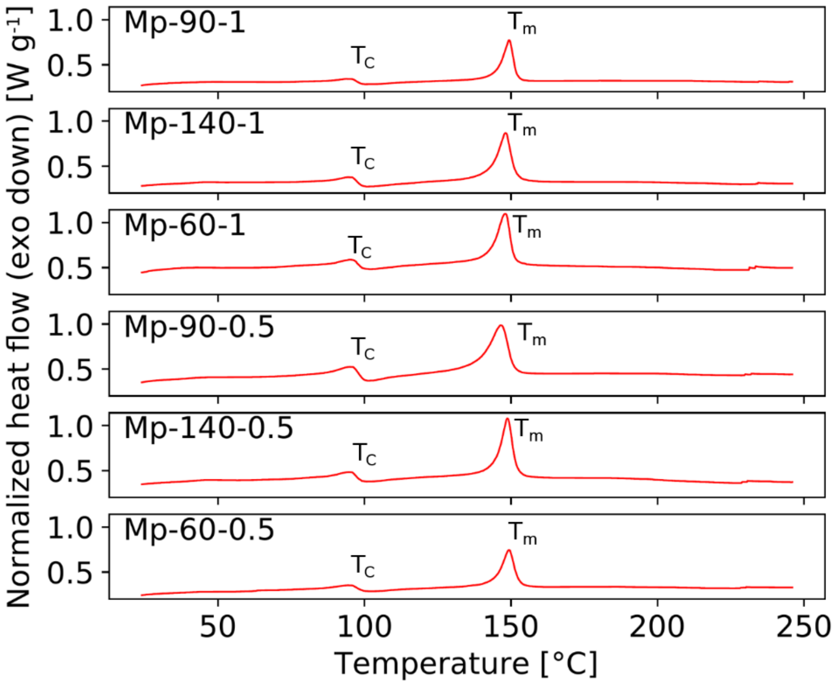


**Figure S8.** Individual DSC thermograms of the MPK-processed membranes

## 5. X-ray diffraction (XRD)

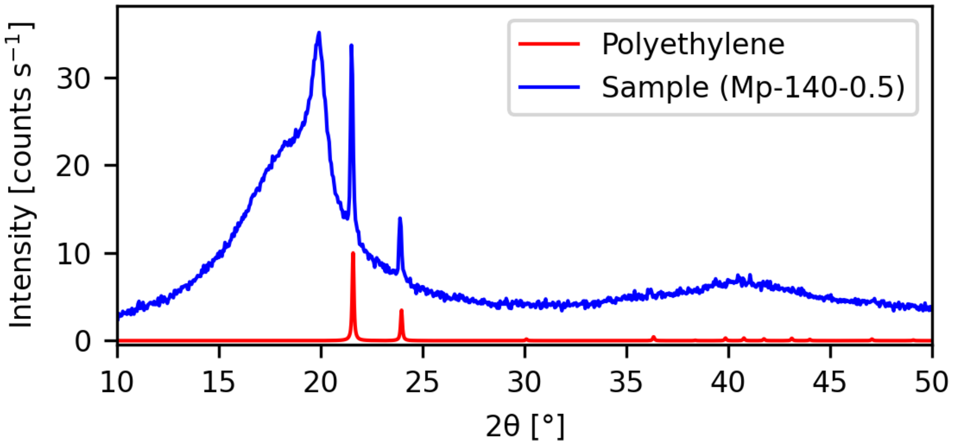


**Figure S9.** Calculated XRD pattern of polyethylene and an example experimental XRD pattern of a specimen with background subtracted. The former was calculated based on the crystal structure model retrieved from the Crystal Structure Database (CSD reference code: QILHUO).[3]

## References


[1] A. Bottino, G. Capannelli, S. Munari, A. Turturro, *Journal of Polymer Science Part B: Polymer Physics* **1988**, *26*, 785–794.

[2] C. M. Hansen, *Hansen Solubility Parameters: A User's Handbook, Second Edition*, CRC Press, Boca Raton, **2007**.

[3] C. R. Groom, I. J. Bruno, M. P. Lightfoot, S. C. Ward, *Acta Cryst B* **2016**, *72*, 171–179.
